# The oral phenotype and dental management in patients with maple syrup urine disease; case report and scoping review

**DOI:** 10.1186/s12903-024-04135-7

**Published:** 2024-03-21

**Authors:** Yazan Hassona, Dua’a Alqaisi, Yara Flaifl, Asma Alkilani

**Affiliations:** 1 Oral Medicine and Special Care Dentistry Faculty of Dentistry, Centre for Oral Diseases Studies (CODS), Al-Ahliyya Amman University, Amman, Jordan; 2 School of Dentistry, The University of Jordan, Amman, Jordan; 3 School of Medicine, The University of Jordan, Amman, Jordan

**Keywords:** Maple syrup, Branched chain amino acids, Ketonuria, Oral, Anesthesia

## Abstract

**Background and objectives:**

The literature about oral manifestations and dental management in maple syrup urine disease (MSUD) is sparse. The aim of this report is to present a new case of MSUD with special emphasis on oral findings and to review the relevant literature.

**Method:**

A case report of a 4-year-old boy with MSUD was described according to the CARE guidelines for describing case reports. Scoping review of relevant literature was performed, according to the PRISMA-ScR guidelines, by searching PubMed, Medline, Embase, and the grey literature for articles describing dental management and/or oral manifestations in MSUD.

**Results:**

The initial search identified 219 articles, but only 4 met the inclusion criteria. Rampant caries and plaque induced gingivitis were the main oro-dental findings in MSUD. Other oral findings included enamel hypoplasia, skeletal abnormalities, and abnormal oral behaviors. Disease-related factors appeared to play a major role in the development of the observed oral phenotype.

**Conclusion:**

Oral health in MSUD seems to be influenced by the reliance on semi-synthetic diet and associated neurocognitive complications. Tailored oral health promotional interventions should be included in the multidisciplinary management of patients with MSUD.

## Introduction

Maple Syrup Urine Disease (MSUD) is an autosomal recessive disorder characterized by an abnormal catabolism of the branched chain amino acids [1]. The disease was first described in 1954 in four siblings with infantile brain oedema, seizures, spasticity, respiratory irregularities, and severe ketoacidosis in association with a strong maple syrup odor to the urine [2]. Later, in 1995, the accumulated materials in the urine were identified as the branched chain amino acids: isoleucine, leucine, and valine [3]. The disease therefore became known as branched-chain ketoaciduria or branched-chain ketonuria.

The abnormal catabolism of the branched chain amino acids is caused by dysfunction of an inner-mitochondrial enzyme complex known as branched-chain alpha-keto acid dehydrogenase (BCKAD) [4, 5]. Enzymatic dysfunction can be caused by various mutations in *BCKDHA*, *BCKDHB*, *DBT, DLD* genes located at chromosomes 19, 6, 1, and 7 respectively; these genes encode the E1α, E1 β, E2 and E3 subunits of the enzyme complex [1, 5–7].

The exact prevalence of MSUD is not known, but figures ranging from 1:200 to 1:185000 have been reported from different parts of the world [8, 9]. In regions with high prevalence, neonatal screening for high blood level of amino acids is routinely performed to allow early detection [7]. Nevertheless, the disease is often diagnosed at late stages, especially in developing countries and in regions where neonatal screening for MSUD is not routinely performed [10].

Currently, there are 5 recognized phenotypes of MSUD: classical, intermediate, intermittent, thiamine-responsive, and E3-deficient types (Table 1) [1, 7, 11]. Symptoms and severity of MSUD vary greatly among affected patients depending on the exact type and the amount of residual enzymatic activity. The classical type is the most common variant of MSUD and is characterized by a distinctive maple syrup odor in urine and sweat that typically appears in the first few days of neonatal life. Without intervention, the disease becomes quickly progressive and leads to brain edema and various manifestations such as irritability, lethargy, feeding difficulties, and metabolic acidosis [7, 12–15]. Neurological symptoms such as spasticity, quadriplegia, and stereotyped movements are particularly common [8, 14, 15].

**Table 1:** Summary of the five main types of MSUD

Type	Age of onset	Affected gene	Clinical features
Classic	Neonatal	*BCKDHA* *BCKDHB* *DBT*	Maple syrup odor of cerumen Poor feeding Irritability, lethargy Opisthotonos Focal dystonia “Fencing,” “bicycling” Obtundation, coma Central respiratory failure
Intermediate	Variable	*BCKDHA* *BCKDHB* *DBT*	Maple syrup odor of cerumen Poor growth Poor feeding Irritability Developmental delays Encephalopathy during illness
Intermittent	Variable	*BCKDHA* *BCKDHB* *DBT*	Normal early growth & development Episodic decompensations that can be severe
Thiamine responsive	Variable	*DBT*	Like the intermediate type
E3 deficient	Variable	*DLD*	Early-onset neurologic phenotype: Hypotonia Developmental delay Emesis Hepatomegaly Lethargy Seizures Spasticity Leigh syndrome Failure to thrive. Hepatic phenotype: Nausea Emesis Hepatomegaly Hepatic encephalopathy

Patients with MSUD require lifelong monitoring of amino acid levels and the use of protein-restricted diet rich in carbohydrates and fat and supplemented with medical amino acids formula [8, 9, 15]. In some patients, hemodialysis or hemofiltration might be needed to remove surplus amino acids, and liver transplantation might offer patients with classical MSUD an effective long-term treatment and may arrest disease progress [8, 16].

The oral health in patients with MSUD might be compromised due to the long-term use of semisynthetic diet and the associated complications such as intellectual disability, obesity, and use of medications. In fact, studies evaluating the oral health status in patients with MSUD are sparse, but increased prevalence of dental caries, dental anomalies and oral infections have been reported [17, 18]. Furthermore, dental management of patients with MSUD might be challenging especially when general anesthesia is needed to facilitate dental treatment. Here, we describe the oral and dental findings in a 4-year-old boy with classical MSUD. Furthermore, we present a scoping review to identify knowledge gaps and outline the scope of literature about oral manifestations and dental management in MSUD.

## Case report

A 4-year-old boy with moderately severe intellectual disability was presented to the special care dentistry clinic regarding the complaints of toothache and sialorrhea. The parents reported that the boy became irritable, agitated, and kept flapping his face and hitting his head during the last few days. The mother reported that the boy became extremely nervous when she attempted to brush his teeth and thought that he might have toothache.

The patient was the first boy for consanguineous parents (maternal cousins) and was born at full-term normal delivery. The medical history was remarkable for moderately severe intellectual disability and generalized hypotonia secondary to MSUD that was diagnosed by a whole exome sequencing performed at the age of 4 weeks. Genetic sequencing revealed the presence of nonsense mutation in the *DBT* gene (NM_001918.2): c.[30G > A] p.[(trp10*)]. The boy was placed on a strict diet (BCAA protein-free food), and high in sugar and carbohydrates. The parents reported episodes of recurrent hypoglycemia that were regularly managed by bolus plain sugar administration.

**Fig. 1:**
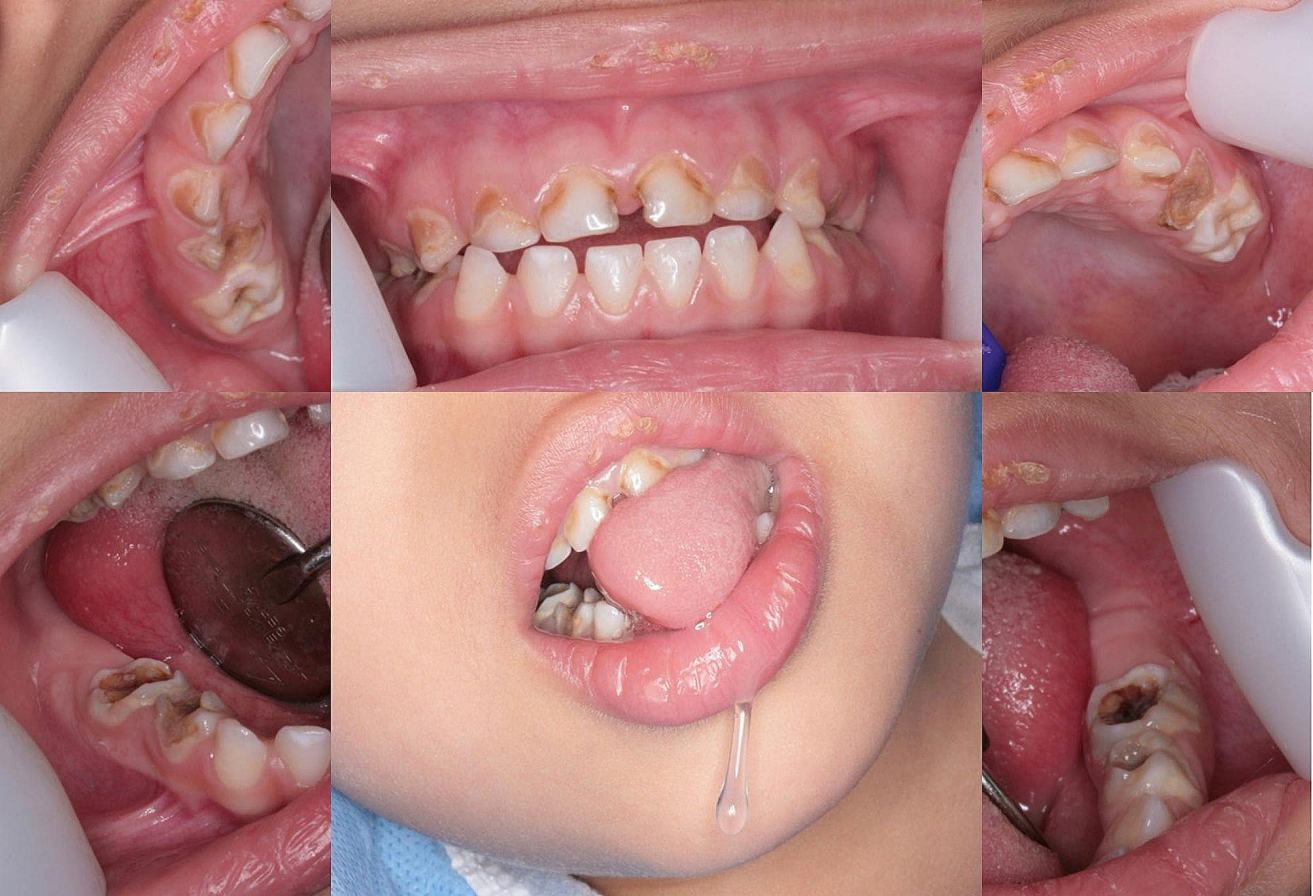
Oral findings in the patient with MSUD included, generalized dental caries involving all posterior teeth and upper anterior teeth, enamel hypoplasia involving the lower anterior teeth, hypotonia with sialorrhea

General examination revealed generalized global development delay (Height of 90 cm; weight of 12 kg), unsteady gait, generalized hypotonia, and impaired expressive and receptive communication. Head and neck examination revealed microcephaly, symmetrical face without swellings or cervical lymphadenopathy, prominent eyelashes, satellite-ears, sialorrhea, and slightly protruded mandible (Fig. 1). Oral examination revealed incompetent lips, and prominent tongue thrust causing anterior open bite. The oral soft tissues were normal, but he had poor oral hygiene, plaque induced gingivitis, and early childhood caries. The lower anterior teeth showed evidence of hypocalcification involving the incisal and middle thirds (Fig. 1).

A comprehensive treatment plan was formulated and involved dietary counseling, oral hygiene instructions, oral physiotherapy, and treatment of tooth decay. The patient was unable to tolerate dental care under local anesthesia and was scheduled for comprehensive dental care under general anesthesia. The parents were informed about the planned procedure and educated about risks and benefits, and written consent was obtained. Pre-anesthetic assessment included neurological and cardiovascular assessment. Evaluation of electrolytes and air blood gases (ABGs) revealed normal results, but the plasma level of branched chain amino acids was elevated (isoleucine 210 µM/L [normal range 26–159 µM/L]; leucine 538 µM/L [normal range 50–264 µM/L]; valine 343 µM/L [normal range 96–566 µML]. At the day of surgery, pre-operative fasting glucose test revealed mild hypoglycemia (70 mg/dl). IV access was established with lactated ringer fluid and naso-endotracheal intubation was performed uneventfully. Anesthesia was induced with sevoflurane inhalation (8%) and cysatracurium (0.15 mg/kg) and was maintained with isoflurane 2%. During surgery, which lasted for 60 min, the patient was hemodynamically stable and dextrose saline 5% was given as boluses of 500 mg twice. Blood glucose was measured twice intra-operatively and readings were comparable to pre-operative readings (73 mg/dl and 80 mg/dl). Recovery went uneventful, but during the postoperative period, the patient had an episode of hypoglycemia (48 mg/dl) and was managed by 2 g bolus of 10% dextrose saline. Dental treatment involved pulpotomy of all molar teeth with direct composite restorations, gingival prophylaxis, and fissure sealant placement. The patient was observed for 6 h during the post-operative period and there were no complications; oral intake was re-established on his BCAA protein-free food. The boy was discharged on the same day of surgery and the parents were educated regarding oral hygiene practices. Oral exercises to minimize sialorrhea were initiated and the patient was referred for dietary counseling. The patient was placed on a three-monthly follow up schedule for oral hygiene reinforcement and fluoride gel application.

## Scoping review

### Materials and methods

A scoping review is defined as a type of knowledge synthesis that uses a systematic and iterative approach to identify and synthesize an existing or emerging body of literature on a given topic. The main reason to perform a scoping review is to map the extent, range, and nature of the literature, as well as to determine possible gaps in the literature on a topic [19]. In the present study, we conducted a scoping review about oral manifestations and dental management in patients with MSUD. This review was conducted following the Joana Briggs Institute guidelines for scoping reviews [19]. A search of the literature was performed using PubMed, Medline, and Embase databases. In addition, the grey literature was searched using Google Scholar and cross referencing of selected articles. The key words used in the search were: (maple syrup urine disease OR branched-chain ketoaciduria OR branched-chain ketonuria) AND (oral OR dental). No time or language restrictions were applied to the database search.

Following removal of duplicates, search results were screened based on their titles and abstract to identify potentially relevant studies. Included studies were selected according to the participants, concept, and context (PCC) criteria (Table 2) [19]. Studies, regardless of the type, describing oral or dental manifestations or discussing oral health care related issues in individuals with MSUD were included in this review.

**Table 2:** The Participant, Concept, Context (PCC) criteria definition in the present review

Criteria	Definition
Participants	Individuals with MSUD, or data derived from these participants.
Concept	Assessment of oral health status of oral health or dental status in individuals with MSUD
Context	Describing individuals with MSUD in the context of oral health diagnosis, management, or prognosis.

### Results and discussion

**Fig. 2:**
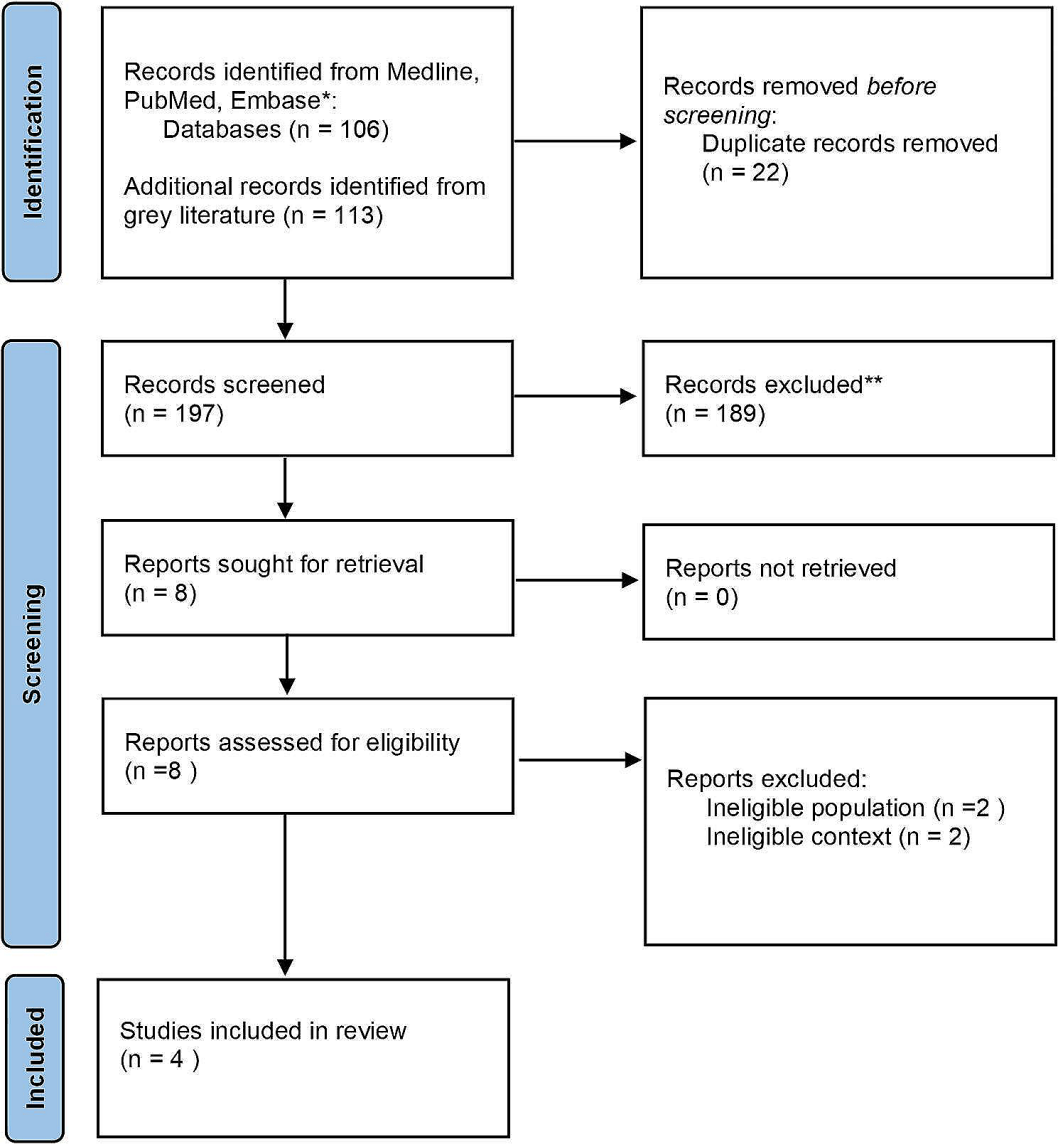
Prisma flow chart for scoping review

An initial search of the databases and the grey literature identified a total 219 of articles. After removal of duplicates and screening of abstracts, a total of 8 articles were evaluated by reading the full text, and only 4 articles met the eligibility criteria and were included in this review (Fig. 2). Three articles were published in English. One article was published with an English abstract and a Japanese full text; the full text was translated, and relevant data were included in the analysis. Findings of the 4 included articles and the findings from our case were categorized under 3 main categories: oro-dental findings; radiographic findings; oral health issues and provision of dental care (Table 3).

**Table 3:** Data extraction table and summary of included studies in the present review

Author	Type of study	No. of participants	Age of participant	Gender of participant	Summary of oral findings	Conclusion
Ballikaya et al., 2021 [17]	Observational	25	9.88 (range from 2 to 28)	10 females; 15 males	Plaque induced gingivitis, dental anomalies and alteration in mandibular morphology, dental caries	Impaired oral health is common among individuals with MSUD. Preventive measures and dental treatment should be included in the multidisciplinary care in MSUD.
Gazit et al., 1989 [18]	Case report	1	14	Male	Elongated and retrognathic mandible, incompetent lips, dental crowding, rampant caries, gingivitis and gingival enlargement,	Oral findings in the described patient cannot be explained solely by the biochemical or metabolic alterations in MSUD.
Fukushima et al., 1986 [20]	Case report	1	5	Female	Micrognathia, dental caries, plaque induced gingivitis	Oral findings are attributed to poor oral hygiene, abnormal eating habit, and restricted diet.
Oelgiesser et al., 2006 [23]	Case report	1	19	Female	Missing teeth due to trauma	Implant supported fixed prosthesis provides functional and esthetic solution for traumatic tooth loss in patients with MSUD.

### Oral findings

The presence of rampant caries was consistently reported in included studies. Fukushima et al., 1986 and Gazit et al., 1989 reported the presence of rampant caries in a 5-year-old girl a 14-year-old boy with classical MSUD respectively [18, 20]. Similar findings were reported in a retrospective study from Turkey where Ballikaya et al., 2021 examined 25 children and young adolescents with MSUD and identified dental caries in 72% of the cases [17]. The mean dmft score was 6.2 and dmfs was 22.26, while the mean DMFT score was 2.66 and DMFS was 5.83. Similarly, our patient demonstrated clinical evidence of early childhood caries. The occurrence of rampant caries in children with MSUD can be attributed to the reliance on semisynthetic milk during early life and to the negative effect of associated nutritional deficiency on tooth development. In addition, children with MSUD often suffer from neurological complications such as hypotonia, spasticity, lack of muscle coordination, and learning disability [1–4]. These abnormalities can increase the risk of dental caries, indirectly, by impairing the normal chewing and swallowing behaviors and affecting the ability to perform oral hygiene measures [21, 22]. The presence of enamel hypoplasia can further increase the risk of dental caries. In fact, enamel defects were reported in more than 50% of the cohort examined by Ballikaya et al., 2021, and in the patient reported by Fukushima et al., 1986 and in our patient [17, 20].

Gingivitis was also consistently reported among included studies, and variable degrees of gingival inflammation were reported in all patients from the included studies including our patient. The mean plaque index score among the cohort examined by Ballikaya et al., 2021 was 2.5 [17]. Gazit et al., 1989 described the occurrence of severe inflammatory gingival enlargement and microscopic evidence of osteomyelitis in a boy with MSUD. The authors, however, failed to provide a biomedical or metabolic explanation to the presence of severe gingival enlargement and bone and soft tissue necrosis, but argued that that chronic destructive inflammatory process in the gingiva and local factors such as poor oral hygiene, heavy calculus formation, and severely decayed teeth could have possibly caused soft tissue and bone necrosis [18].

Skeletal abnormalities reported among patients with MSUD included elongated faces, incompetent lips, crowding, small dental arches, and abnormal mandibular position (i.e. prognathism or retrognathism) [17, 18, 20, 23]. The exact cause for these skeletal abnormalities is not known, but it seems that the presence of hypotonia and lack of muscle coordination combined with soft artificial diet could be a major contributor to the observed skeletal phenotype [24–28].

### Radiographic findings

Data about radiographic evaluation in patients with MSUD was available in two studies [17, 20]. Fukushima et al., 1986 reported no abnormal dental radiographic findings in their reported patients. However, Ballikaya et al., 2021 examined 12 patients using orthopantogram, and identified dental anomalies in 58% of the examined patients. Reported abnormalities included Turner hypoplasia, aberrant mandibular premolar, taurodontism, flattening of the mandibular condyle, hyperplasia of the coronoid process, and bifid mandibular canal [17]. It seems difficult to draw any fresh conclusion regarding radiographic findings in MSUD because of the limited number of evaluated patients and the lack of standardized radiographic assessment protocol in the study of Ballikaya et al., 2021.

### Oral health issues and provision of dental care

Although the number of included studies is limited, all reported patients exhibited generalized dental caries and a variable degree of gingival inflammation. These findings suggest that dental caries and plaque induced gingivitis are the major oral health related issues in patients with MSUD. It seems difficult to adequately interpret the findings of dental caries and gingivitis in patients with MSUD because of the limited number of included patients. Nevertheless, comparing these findings with studies evaluating oral health in a relatively similar metabolic disease (i.e. phenyl ketonuria [PKU]) might provide some insight on oral health in patients with MSUD. Most studies in children with PKU have revealed a similar or lower prevalence of caries compared to healthy children [29–31]. A low rate of caries despite a highly cariogenic diet might result from high phenylalanine (the accumulating amino acid in PKU), which has been considered to possibly act as a factor that limits the growth of plaque microorganisms [31]. It seems that the high prevalence of dental caries in MSUD might be attributed to the oral health behaviors, diet, and concomitant diseases such as epilepsy or intellectual disorders. In fact, it was reported that intellectual performance of children with MSUD patients was significantly lower than that of a matched cohort of early treated PKU patients [32]. Oral healthcare professionals dealing with MSUD therefore should perform adequate assessment to disease-related factors that can contribute to the increased prevalence of dental caries and gingivitis such as lip incompetence, mouth breathing/tongue thrusting behaviors, hypotonia and slow chewing cycle, and abnormal swallowing pattern [24–28]. Furthermore, dietary assessment and counseling should be an integral part of any oral health promotional program directed at patients with MSUD.

In addition to caries and gingivitis, sialorhoea and abnormal chewing/swallowing behaviors seem to be important oral health issues in MSUD. Multidisciplinary care involving oral health education, physiotherapy, and chewing exercises should be considered essential in patients with MSUD.

Individuals with MSUD might suffer from traumatic oral and dental injuries because of associated neurological complications such as seizures, spasticity, and lack of muscle coordination. In fact, Dan et al., 2006 reported the placement of dental implant in a 19-year-old girl with MSUD who sustained avulsion of an upper anterior tooth following seizure episode [23]. Fixed prosthesis seems to be preferable in patients with MSUD to minimize the risk of aspiration associated with hypotonia, learning disability, and seizure episodes.

The provision of dental care in patients with MSUD seems to be challenging because of the presence of learning disability, the lack of muscle coordination, and the rampant dental disease. Dental care under general anesthesia therefore might be necessary. Dental care under general anesthesia was described in 3 studies [18, 20, 23]. However, details of pre-anesthetic assessment, administrations of general anesthesia, and post-operative recovery were not adequately described. Patients with strict artificial diet are more vulnerable to nutritional deficiency which might complicate the provision of dental care under general anesthesia. Pre-operative assessment should include evaluation of hemoglobin level, branched chain amino acid levels, and plasma electrolytes level. Neurological assessment of disease severity and susceptibility to seizure episode should also be considered during the pre-operative assessment. In addition, patients with MSUD, especially the very young ones, are susceptible to hypoglycemia, which was evident in our case. Therefore, regular monitoring of blood glucose level during dental treatment and in the early post-operative period is recommended.

## Conclusion

Oral manifestations in MSUD are rarely reported in the literature, and solid conclusions are difficult to obtain from this review due to the small number of included articles. Nevertheless, dental caries and plaque induced gingivitis are the main oral health related issues in individuals with MSUD. Reliance on semi-synthetic diet, associated neurological complications, lack of oral hygiene practices, abnormal oral behaviors, and enamel hypoplasia seem to be the major contributing factors. Tailored oral health educational programs should be considered an integral part of the multidisciplinary management of patients with MSUD. Adequate knowledge and training are required for the proper delivery of tailored oral healthcare for individuals with special healthcare needs including those with MSUD. Furthermore, promotion campaigns for oral health in individuals with special healthcare needs are necessary to improve the awareness of parents and caregivers [33–35].

Further studies describing the oro-dental findings in patients with MSUD are encouraged to help better define the “oral” phenotype in this disease, to identify the major oral health related concerns, and to develop better understanding to the provision of dental care of affected patients. Areas of knowledge gap in the scope of oral health in MSUD are numerus and include the effect of the semisynthetic diet on tooth development, oral health interventions in children with MSUD, the oral phenotype in older patients, and the impact of dental diseases of MSUD on the overall health and quality of life in affected patients.

## Data Availability

Data are available from corresponding author on reasonable request.
